# Higher glucose level can enhance the *H. pylori* adhesion and virulence related with type IV secretion system in AGS cells

**DOI:** 10.1186/s12929-014-0096-9

**Published:** 2014-10-09

**Authors:** Shew-Meei Sheu, Hsin Cheng, Cheng-Ye Kao, Yao-Jong Yang, Jiunn-Jong Wu, Bor-Shyang Sheu

**Affiliations:** Institute of Basic Medical Sciences, College of Medicine, National Cheng-Kung University, Tainan, Taiwan; Department of Medicine, College of Medicine, National Cheng-Kung University, Tainan, Taiwan; Department of Pediatrics, College of Medicine, National Cheng-Kung University, Tainan, Taiwan; Department of Medical Laboratory Science and Biotechnology, College of Medicine, National Cheng-Kung University, Tainan, Taiwan; Department of Internal Medicine, National Cheng Kung University Hospital, College of Medicine, National Cheng Kung University, #138 Sheng Li Road, Tainan, Taiwan

**Keywords:** Glucose, *Helicobacter pylori*, CagA

## Abstract

**Background:**

Hyperglycemia increases the risk of gastric cancer in *H. pylori*-infected patients. High glucose could increase endothelial permeability and cancer-associated signaling. These suggest high glucose may affect *H. pylori* or its infected status.

We used two strains to investigate whether *H. pylori* growth, viability, adhesion and CagA-phosphorylation level in the infected-AGS cells were influenced by glucose concentration (100, 150, and 200 mg/dL).

**Results:**

The growth curves of both strains in 200 mg/dL of glucose were maintained at the highest optimal density after 48 h and the best viability of both strains were retained in the same glucose condition at 72 h. Furthermore, adhesion enhancement of *H. pylori* was significantly higher in 200 mg/dL of glucose as compared to that in 100 and 150 mg/dL (p < 0.05). CagA protein also increased in higher glucose condition. The cell-associated CagA and phosphorylated-CagA was significantly increased in 150 and 200 mg/dL of glucose concentrations as compared to that of 100 mg/dL (p < 0.05), which were found to be dose-dependent.

**Conclusion:**

Higher glucose could maintain *H. pylori* growth and viability after 48 h. *H. pylori* adhesion and CagA increased to further facilitate the enhancement of cell-associated CagA and phosphorylated CagA in higher glucose conditions.

## Background

*Helicobacter pylori* infection in the human stomach causes chronic inflammation, leading to peptic ulcers and gastric malignancy [1-3]. Other cofactors may be involved in the *H. pylori* related gastric carcinogenesis, because not all *H. pylori*-infected patients develop gastric cancer. Some reports have shown that there is a significantly higher prevalence of *H. pylori* infection in patients with type 2 diabetes mellitus (DM), who also have a lower eradication rate [4-6]. In particular, a higher fasting plasma glucose level in *H. pylori-*seropositive patients may correlate with an increased risk of gastric cancer up to a nearly 3.5-4.2 fold increase [7]. These data suggest that hyperglycemia may be an important cofactor to influence *H. pylori* mediated gastric carcinogenesis [8].

To establish *H. pylori* colonization and chronic inflammation at the gastric epithelium, adherence can serve as the first step of infection and then deliver effectors to induce inflammation [9-11]. The major adhesion, blood group antigen binding adhesin (BabA), binds to Lewis b (Leb) and related ABO antigens to initiate bacterial adhesion and stimulate IL-8 secretion [12-14]. Moreover, cytotoxin-associated gene A antigen (CagA), an onco-protein, is translocated into gastric epithelial cell through type IV secretion system of *H. pylori* [15-18]. The tyrosine in the EPIYA motif of CagA C-terminal could be phosphorylated [19] and thus can change the cell morphology, increase cell motility, and promote cell proliferation [20-23].

Hyperglycemia leads into an increased risk of gastric cancer in the *H. pylori*-infected patients [7]. Moreover, high glucose can enhance cancer-associated Wnt/β-catenin signaling [24]. These results suggest a link between hyperglycemia*, H. pylori* and gastric cancer. High glucose could also increase endothelial permeability and altered basement membrane composition and structure [25,26], which may make *H. pylori* infection occur in a high glucose condition, such as the patients with hyperglycemia. Therefore, it is worth investigating whether or not glucose can influence the expressions of *H. pylori* virulence to promote carcinogenesis. This study is original to illustrate that higher glucose concentrations can promote bacterial growth of *H. pylori* isolates, facilitate bacterial adhesion to gastric epithelial cells, and up-regulate the expression of CagA protein to facilitate more virulence related to type IV secretion system.

## Methods

### Assessment to the *H. pylori* growth under different glucose levels

Strain J99 and ATCC 43504 were purchased from ATCC and stored at –70°C in BHI with 30% glycerol until testing was conducted. *H. pylori* strains were cultured on CDC anaerobic blood agar (BBL, Microbiology Systems, Cockeysville, MD, USA). When determining growth curve, *H. pylori* strains were cultured in Brucella broth containing 10% horse serum and different concentration of glucose (100, 150, 200 mg/dL) at 37°C for 3 days in a microaerophilic conditions, with shaking at 50-52 rpm. The optical density (OD) at 600 nm of bacterial growth was evaluated at 20, 48, and 72 h. Bacterial viability was detected at 48 and 72 h of growth curve and analyzed by serious dilution to count colony formation unit (CFU) per ml.

### Co-culture of *H. pylori* & AGS cells under different glucose levels

The human gastric adenocarcinoma cell line, AGS, was purchased from Food Industry Research and Development Institute in Taiwan and was grown in the cell medium, Ham’s F-12 medium (GIBCO BRL, Grand Island, NY) containing 10% FCS. The cells were sub-cultured every second to third day. To conduct the *H. pylori* adhesion assays in AGS cells, the AGS cells (5.5 × 10^5^/well) were seeded to one well of 6-well plates for 22 h and then replaced the cell medium to cell medium supplemented with different concentrations (100, 150, 200 mg/dL) of glucose for 3.5-4 h. After washing with the mixture of F-12 medium and 1× PBS (1:1) to the AGS cells cultivated in medium with specific concentrations of glucose, the serum-free F-12 medium (SFM) with the same glucose concentration were added again. As well, the both *H. pylori* strain J99 and 43504 were cultured for 20 hours in Brucella broth including 10% horse serum under three glucose concentrations, including 100, 150, 200 mg/dL, respectively. The bacteria shall be washed twice with the SFM containing the same glucose contraction before adding to cells. After washing, these bacterial suspensions in the SFM will be then applying to the well that contained AGS cells with the same concentration of glucose treatment at Multiplicity of infection (MOI) as 30-40. In each glucose level, there should be co-cultured with *H. pylori* and AGS cells for two wells, including the first one for adhesion assay, the second one for the detection of CagA phosphorylation. The first well was washed three times with the SFM 30 min later to detect bacterial adhesion and the second well was incubated for 4 h to detect CagA phosphorylation.

### *H. pylori* adhesion assay to AGS cells under different glucose levels

In order to calculate the percentage of bacterial adhesion, lysate of *H. pylori* adhering to AGS cells and the original suspension of *H. pylori* adding to adhesion assays were seriously diluted respectively and grown on CDC plates to count bacterial CFU. The adhesion CFU divided by the original CFU is the adhesion percentage of *H. pylori*.

### Western blotting for detection H. pylori BabA, CagA and CagA phosphorylation

The bacterial suspension adding to detect adhesion ability under different glucose concentration was also collected to determine protein concentration. An equal amount of bacterial protein (1.5-2 μg) was used to detect the expression of BabA and CagA by performing western blotting. Moreover, the well of co-cultured *H. pylori* and AGS cells incubated for 4 h was washed 3 times to collect cell lysates for detection of CagA and CagA phosphorylation. Antibodies (Ab) used in western blotting included the anti-BabA as applied in our previous report [27], anti-phospho-tyrosine (PY99) (Santa Cruz Biotechnology), anti-CagA (Santa Cruz Biotechnology) and anti-beta Actin (Minipore) Abs.

### Statistics

The statistical analysis was performed by using a paired *t* test. The differences were considered to be significant at *p* < 0.05.

## Results

### Effect of glucose level on H. pylori growth curve and viability

**Figure 1:**
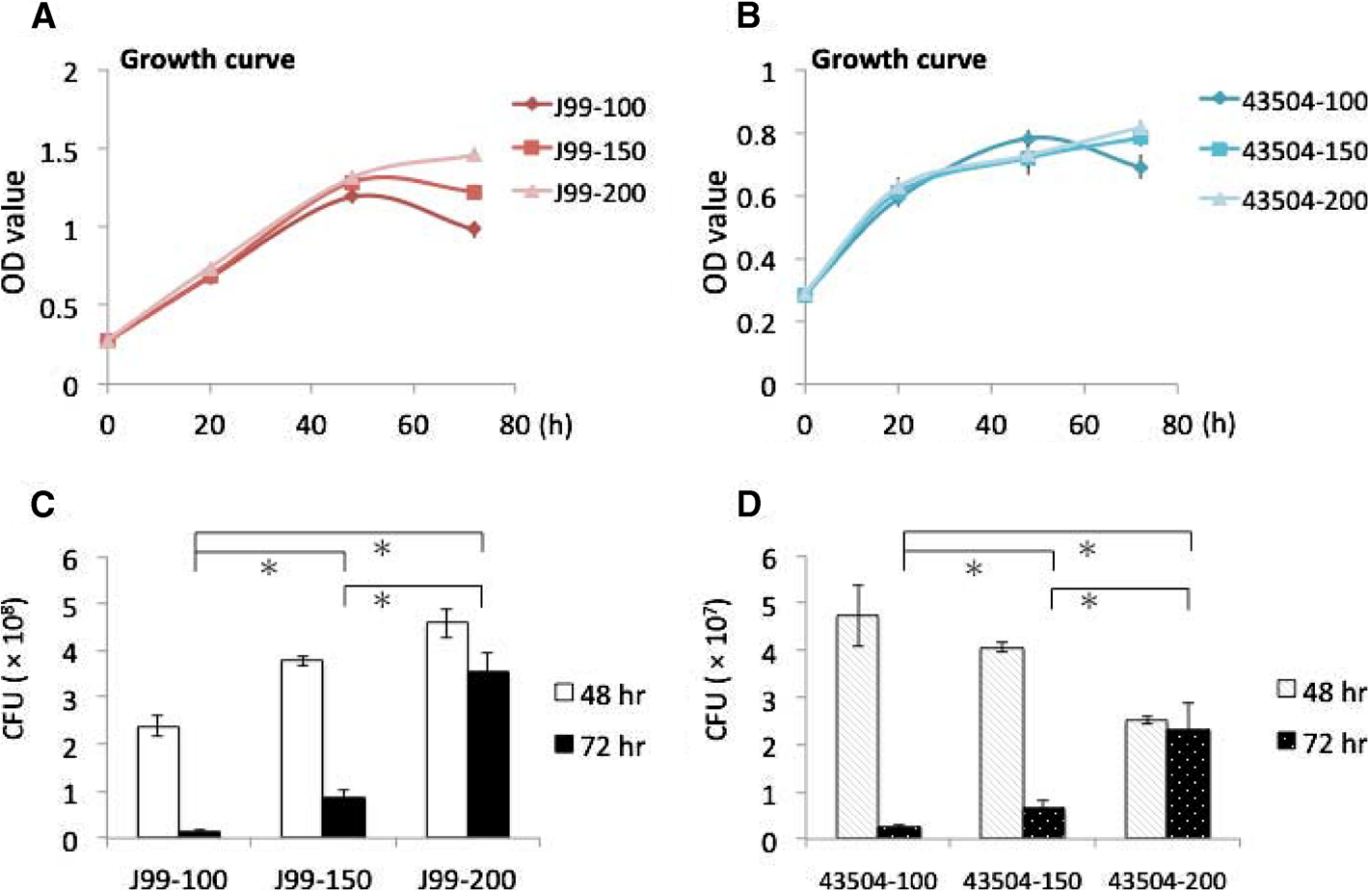
***H. pylori*** **growth and viability was increased under higher glucose level after 48 hr. (A, B)** Strain J99 and 43504 grew in Brucella broth containing 10% horse serum and different level of glucose (100, 150, 200 mg/dL). The optical density (OD) at 600 nm of bacterial growth was evaluated during 72 h. **(C, D)** Colony formation unit (CFU) of strain J99 and 43504 was determined at growth curve of 48 and 72 h. * indicated p < 0.05 (paired *t* test).

Strain J99 and 43504, grown in broth containing three concentrations of glucose (100, 150, 200 mg/dL), were applied to detect the growth curve during 72 h (Figure 1A and B). Optical density of strain J99 at 200 mg/dL were kept on increasing at 72 h, however, OD value was decreased in 100 and 150 mg/dL of glucose conditions at the same time (Figure 1A). The significant difference between 3 growth curves of strain J99 was observed at 48 h (J99-100 vs. J99-150, p < 0.05; J99-100 vs. J99-200, p < 0.05; J99-150 vs. J99-200, p > 0.05) and 72 h (J99-100 vs. J99-150, p = 0.05; J99-100 vs. J99-200, p < 0.05; J99-150 vs. J99-200, p < 0.05). Growth of strain 43504 in higher glucose concentrations (150 and 200 mg/dL) had the trend of higher optimal density, as compared to low glucose (100 mg/dL), except 48 h. Besides strain 43504 at 48 h, *H. pylori* viability was increased by the increasing glucose concentration (Figure 1C and D). Especially at 72 h, the viability of two strains cultured in 150 and 200 mg/dL were consistently increased as compared to that in 100 mg/dL (p < 0.05). Viability showed 3-4 fold increases in 200 mg/dL as compared to that in 150 mg/dL (p < 0.05).

### Higher glucose level treated to *H. pylori* isolates enhance the bacterial adhesion

**Figure 2:**
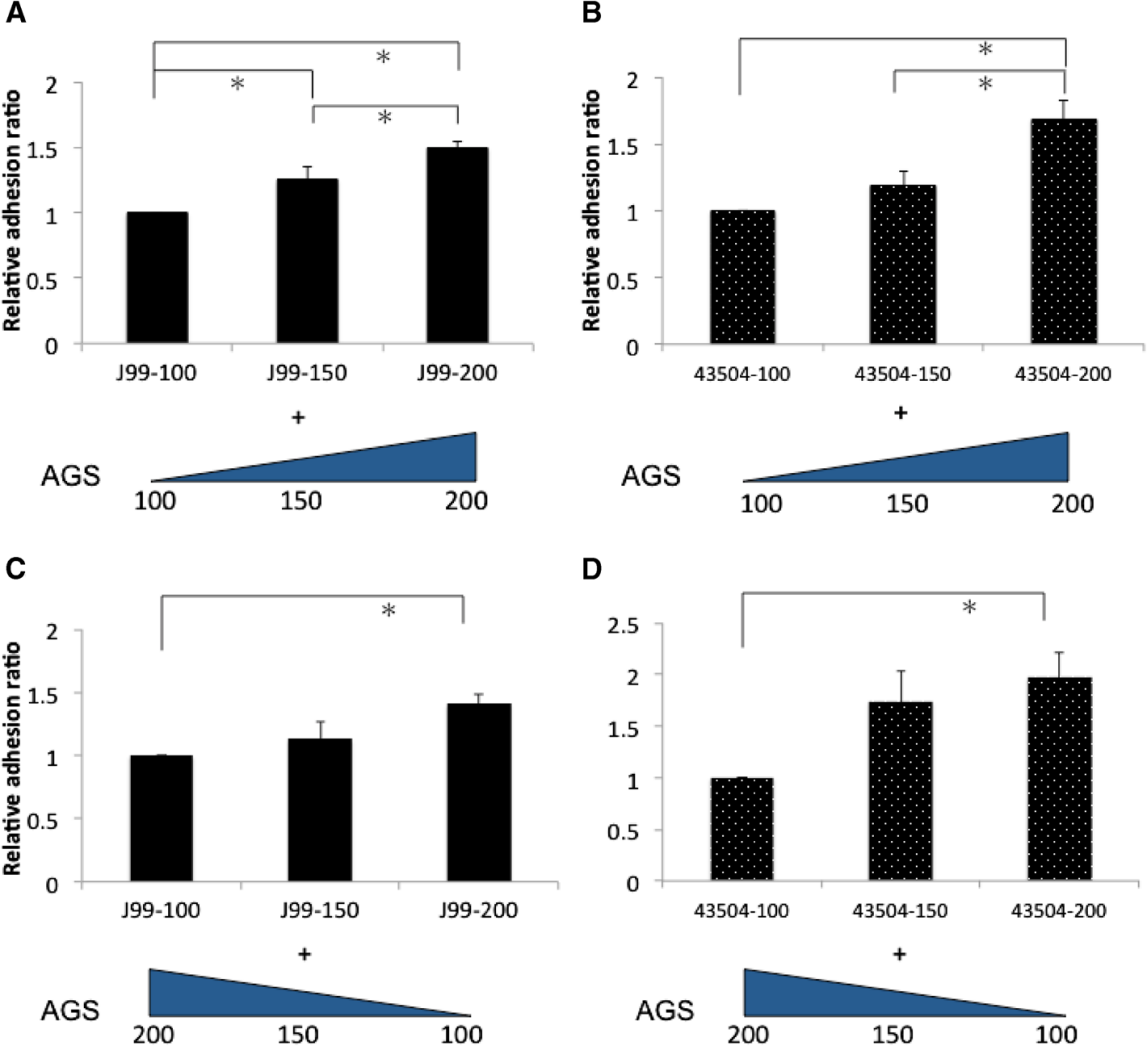
**Adhesion ability of** ***H. pylori*** **increased under the treatment of higher glucose concentration.** Strain J99 and 43504 growth in different level of glucose (100, 150, 200 mg/dL) was added to AGS cells with the pretreatment of the same glucose concentration **(A, B)** or the pretreatment of contrary concentration (200, 150, 100 mg/dL) **(C, D)**. Relative adhesion ratio indicates that the adhesion of *H. pylori* in 100 mg/dL of glucose serve as the reference, and the bacterial adhesion value in 150 and 200 mg/dL of glucose was divided by the value of reference. *indicated a significant difference between the treatment of different glucose concentrations (paired *t* test, *p* < 0.05).

With the treatment of 200 mg/dL of glucose to *H. pylori* and AGS cells, the adhesion ability of strain J99 and 43504 was significantly enhanced as compared with that of 150 and 100 mg/dL of glucose treatment (Figure 2A and B). In order to test whether cellular factors play some role in adhesion enhancement, strain J99 and 43504 growth in 100, 150, and 200 mg/dL of glucose adhered to AGS cells with pretreatment of with contrary concentration (200, 150, 100 mg/dL), respectively (shown in Figure 2C and D). Nevertheless, the adhesion abilities within each strain were similarly disclosed with an increasing trend based on the glucose level treated to the bacteria (strain J99 on Figure 2A and C; strain 43504 on Figure 2B and D).

To check that the effect of glucose on increasing bacterial growth and adhesion not due to change osmolality, we used L-glucose without biological activity to assess the same experiment. Our data confirmed L-glucose did not increase OD value at 72 h. Additionally, the adhesion ability of both strains in either 150 or 200 mg/dL of glucose were similar to that in 100 mg/dL (data not shown).

### Effect of glucose level on H. pylori type IV secretion system-related virulence

**Figure 3:**
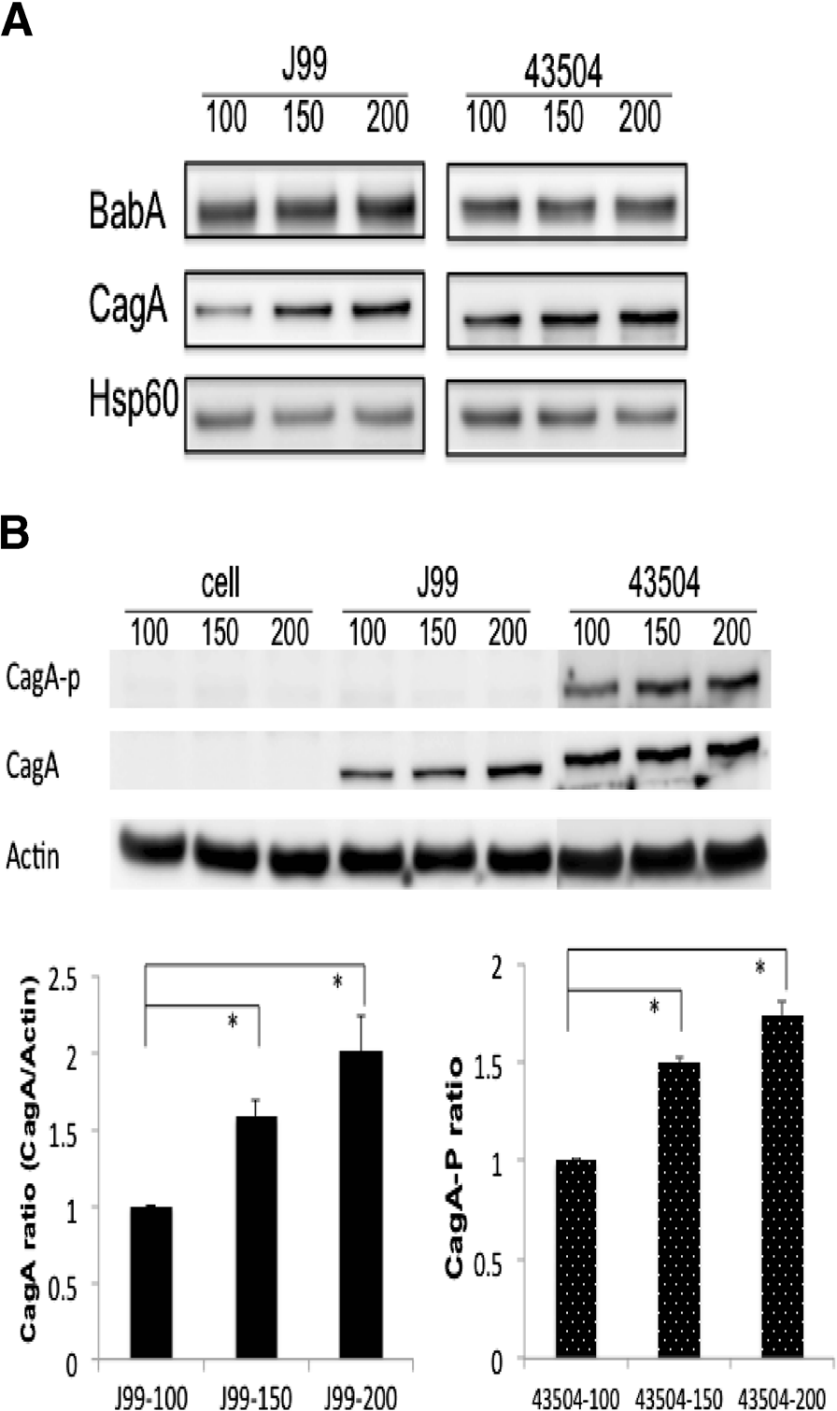
**Figure 3** ***H. pylori*** **CagA and phosphorylated CagA increased in higher glucose concentration. (A)** BabA and CagA expression of *H. pylori* grown in three glucose concentrations. **(B)** The level of cell-associated CagA and phosphorylated CagA after *H. pylori* infecting AGS cells for 4 h. Thee glucose concentrations were 100, 150 and 200 mg/dL, respectively. *indicated a significant difference between the treatment of different glucose concentrations (paired *t* test, *p* < 0.05).

BabA expression of *H. pylori*, in either strain J99 or 43504, was similar among three glucose concentrations including 100, 150, and 200 mg/dL. However, for either one isolates, the expression of CagA can be increased as the bacteria exposed to glucose concentration which was raised from 100 to 200 mg/dL (Figure 3A). When *H. pylori* infected to AGS cells, the cell-associated CagA of strain J99 presented with a significant stepwise increase by the glucose treatment in level up to 150 and 200 mg/dL (p < 0.05), as compared to the glucose treatment as 100 mg/dL (Figure 3B). Also in Figure 3B, the cell-associated CagA of strain 43504 had the similar scenario to that of strain J99. Strain 43504 had a significantly increased trend of phosphorylated CagA levels ranking in order parallel to the elevation of glucose levels from 100, 150, and up to 200 mg/dL (p < 0.05, Figure 3B).

## Discussion

The identification of cofactors involved in *H. pylori-*related gastric carcinogenesis is important due to the fact that not all *H. pylori*-infected patients developing gastric cancer. Higher fasting plasma glucose levels in *H. pylori-*seropositive patients significantly increased the risk of gastric cancer, suggesting that hyperglycemia may contribute to *H. pylori-*related gastric carcinogenesis [7,8]. The possible mechanisms by which hyperglycemia affects *H. pylori*-related gastric carcinogenetic process include: (1) glucose itself may activate Wnt/β-catenin pathway or increase reactive oxygen species to induce DNA damage, which contribute to *H. pylori* pathogenic effect; (2) hyperglycemia influences *H. pylori* and *H. pylori*-related infectious status or stimulates its carcinogenic effect. However, at present, the detailed mechanism is remains unclear. In the present study, we demonstrate that higher glucose could maintain *H. pylori* growth and viability after 48 h. *H. pylori* adhesion and CagA expression was further increased to facilitate the enhancement of cell-associated CagA and phosphorylated CagA in higher glucose conditions. These results support the second possible mechanism.

Reynolds and Pann demonstrated that glucose enhances *H. pylori* growth in a defined medium [28]. Albertson et al. [29] found that the presence of glucose did not show growth enhancement on *H. pylori*. This discrepancy may be due to strain variations and detection methods utilized. In the study of Albertson et al, growth and yield of *H. pylori* (numbers of CFU per milliliter) was shown to have no difference by adding 0.2 or 1.0% glucose (200 or 1000 mg/dL) to the defined medium supplemented with 0.5% BSA. This could be due to the high bacterial density (10^7^ CFU/ml) at the beginning of inoculation. Glucose was rapidly consumed and could not provide for further growth because of growth and yield only increased before 20 h. In our study, we found that higher glucose concentrations (200 mg/dL) could keep up the growth curve detected by OD at 600 nm after 48 h of inoculation (Figure 1A and B). Moreover, the viability of *H. pylori* at the same concentration of glucose was obviously higher than that of 100 or 150 mg/dL at 72 h (Figure 1C and D). This data was supported by Albertson et al. [29]. They found that viability of *H. pylori* in the presence of glucose obviously retains after inoculation of 2 days, as compared to non-glucose addition. We further provide the result that glucose can enhance *H. pylori* viability with a dose-dependent effect at 72 h (Figure 1C and D).

Whether or not an infection rate of *H. pylori* is higher in DM patients remains controversial. Some studies report that the prevalence of *H. pylori* in DM patients shows no significant difference as compared to controls [30,31]. In these articles, only the *H. pylori* infection rate was studied, but no more detailed analysis was conducted to trace the *H. pylori* density-related histology or virulence severity. Our study revealed that *H. pylori* adhesion was enhanced in higher glucose concentrations (Figure 2A and B). We provide data supporting the possibility that glucose may influence *H. pylori* density colonizing on the gastric epithelium. *H. pylori* colonization could further interact with gastric epithelium to induce gastric inflammation [32,33]. In diabetic patients, *H. pylori* infection was significantly associated with chronic gastritis, but not in dyspeptic patients [34].

This study should be considered as being original to illustrate that bacterial adhesion to gastric epithelial cells can be enhanced by a higher glucose condition. *H. pylori* growth in 100, 150 and 200 mg/dL of glucose is shown to have an increasing trend after being added to AGS cells pretreated with contrary concentrations of glucose (Figure 2C and D). It indicates that bacterial factors may play a more important role in increasing adhesion. When we analyzed BabA expression of *H. pylori* growth in three different glucose conditions, it did not show significant differences (Figure 3A). However, CagA expression was elevated as *H. pylori* growth in the increasing concentration of glucose (Figure 3A). Cell-associated CagA of strain J99 and phosphorylated CagA of strain 43504 were significantly increased in higher glucose conditions of infection (150 and 200 mg/dL) (Figure 3B). Therefore, the enhancement of cell-associated or phosphorylated CagA could be due to the elevated adhesion ability and CagA expression of *H. pylori* in higher glucose conditions (Figures 2A, B and 3A).

Ishijima et al. [33] demonstrated that BabA-mediated adherence increases the effectiveness of *H. pylori* type IV secretion activity, implying the promoting role of *H. pylori* adhesion in type IV secretion activity. Moreover, phosphorylation of CagA is essential for induction of gastrointestinal neoplasm in transgenic mice [35]. Due to the elevated phosphorylated CagA in higher glucose conditions, it may further contribute to facilitate a more evident gastric carcinogenesis in the *H. pylori*-infected DM patients.

## Conclusions

In summary, we provide *in vitro* evidence that *H. pylori* growth, viability, CagA and phosphorelated CagA after infecting gastric epithelial cells could be enhanced in higher glucose condition, supporting that hyperglycemia could be a cofactor to increase a risk of *H. pylori* related gastric carcinogenesis. The evidence would indicate that it is important to conduct large-scale *H. pylori* screening and eradication to control the increased risk of gastric cancer for DM patients, especially in high *H. pylori*-infected or gastric cancer prevalent countries.
